# ERRATUM: Intratumoural aldosterone and CYP11B2 expression levels among different genotypes of aldosterone producing tumours

**DOI:** 10.1530/EC-25-0070e

**Published:** 2026-02-25

**Authors:** Tobias Åkerström, Branislav Klimàcek, Matilda Annebäck, Olov Norlén, Peter Stålberg

**Affiliations:** Department of Surgical Sciences, Uppsala University Hospital, Uppsala, Sweden

The authors and journal apologise for an error in the above paper, which appeared in volume 14, part 6, article ID e250070. The error relates to a sentence in the ‘Aldosterone measurement’ section given on page 4, in which ‘lower levels of tissue aldosterone’ was stated. This should have stated ‘higher levels of tissue aldosterone’. The corrected sentence is given in full below:

The original stated:

‘Tumours with mutations in *ATP1A1*, *ATP2B3* and *CACNA1D* exhibited significantly lower levels of tissue aldosterone compared to those with *KCNJ5* mutations …’

This should have stated:

‘Tumours with mutations in *ATP1A1*, *ATP2B3* and *CACNA1D* exhibited significantly higher levels of tissue aldosterone compared to those with *KCNJ5* mutations …’

